# Lifting Hofmeister’s Curse: Impact of Cations on Diffusion, Hydrogen Bonding, and Clustering of Water

**DOI:** 10.1021/jacs.3c09421

**Published:** 2023-12-21

**Authors:** Mario González-Jiménez, Zhiyu Liao, Elen Lloyd Williams, Klaas Wynne

**Affiliations:** School of Chemistry, University of Glasgow, Glasgow G12 8QQ, U.K.

## Abstract

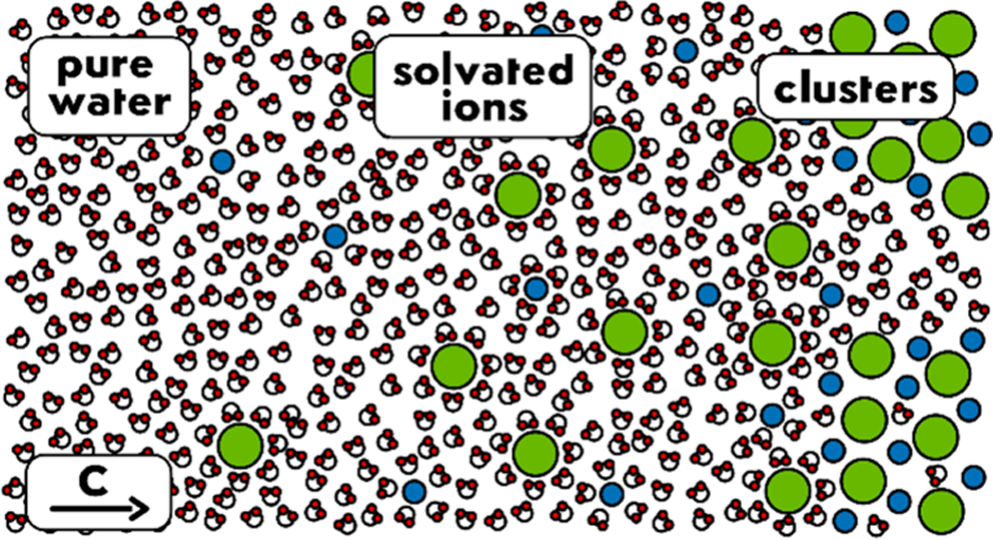

Water plays a role in the stability, reactivity, and dynamics of the solutes that it contains. The presence of ions alters this capacity by changing the dynamics and structure of water. However, our understanding of how and to what extent this occurs is still incomplete. Here, a study of the low-frequency Raman spectra of aqueous solutions of various cations by using optical Kerr-effect spectroscopy is presented. This technique allows for the measurement of the changes that ions cause in both the diffusive dynamics and the vibrations of the hydrogen-bond structure of water. It is found that when salts are added, some of the water molecules become part of the ion solvation layers, while the rest retain the same diffusional properties as those of pure water. The slowing of the dynamics of the water molecules in the solvation shell of each ion was found to depend on its charge density at infinite dilution conditions and on its position in the Hofmeister series at higher concentrations. It is also observed that all cations weaken the hydrogen-bond structure of the solution and that this weakening depends only on the size of the cation. Finally, evidence is found that ions tend to form amorphous aggregates, even at very dilute concentrations. This work provides a novel approach to water dynamics that can be used to better study the mechanisms of solute nucleation and crystallization, the structural stability of biomolecules, and the dynamic properties of complex solutions, such as water-in-salt electrolytes.

## Introduction

Water is the basis of life, not only because it is the medium in which biological processes take place but also because it actively participates in them. One of the best examples is the phenomenon of solvation, in which water molecules facilitate the transport of ions,^1^ influence chemical reactions,^2^ and support and determine the structure of proteins, nucleic acids, etc.^3,4^

The action of water in all of these processes is, however, influenced by the salts present in the aqueous solution. This influence was first described by Hofmeister in 1888, who classified ions according to their ability to increase or decrease the solubility of egg whites in water.^5^ This list is known today as the lyotropic or Hofmeister series. The order of the cations in this series depends largely on their charge density.^6^ If a small or highly charged ion is added to the solution, the water molecules prefer to solvate it rather than the protein and therefore reduce the solubility of the protein (salting-out effect). These ions are also known as structure makers or, from Greek, *kosmotropes* because they tend to order the water molecules around them. A common confusion in the literature is to think that these names refer to the effect of the ions on all of the water molecules in the solution rather than the solvating water molecules.

Ions with low charge density are called structure breakers or *chaotropes* because of their opposite properties. However, despite their name, their electrostatic effect on water is small, and their main contribution to disrupting the structure of water is due to their large masses and sizes. In their solvation layer, the interactions of water molecules are dominated by water–water hydrogen bonding.^7^ They interact weakly with the nonpolar parts of a protein and increase its solubility (salting-in effect).

The order of the cations in the Hofmeister series depends on the properties of the solute in the solution (either protein or any other nonelectrolyte) and the affinities between the solution components.^8^ If this solute is nonpolar, there is a correlation between the salting-out intensity and the reduction in volume that the solution undergoes when the salt is added, indicating that the structural factors of the solution also play a role in the effect.^9^ In this case, the alkaline ions, ranked according to their ability to alter the solubility of the solute, follow the sequence Cs < Rb ≈ Li < K < Na. If the solute is polar, the sequence is similar, but the size and polarity of the solute will influence the magnitude of the effect. In addition, if the solute is basic, the charge of the anion has the greatest influence, and if it is acidic, the charge of the cation has the greatest influence on the effect, and alkali ions follow the sequence Cs < Rb < K < Na < Li.^10^

**Figure 1:**
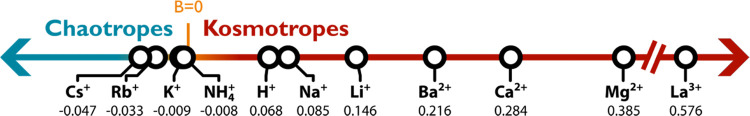
Chaotropic and kosmotropic effects of ions as expressed through the Jones–Dole *B*-coefficient. *B* coefficients of the ions used in this work, shown on the Hofmeister scale.^15^

The Hofmeister series can help to understand how different ions in a solution can influence its viscosity according to their relative abilities to induce the structuring of water.^11^ Chaotropic ions tend to reduce viscosity, while kosmotropic ions increase viscosity. The increase or decrease in viscosity in water due to added salt is given by the Jones–Dole equation,^12^
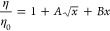
where *x* is the salt concentration, η is the viscosity of the solution, and η_0_ is the viscosity of pure water at the same temperature. *A* and *B* are empirical constants. *A* can be explained by the Debye–Hückel theory as being due to counterion screening at low ion concentrations. The constant *B* is related to the degree of water structuring and is positive for kosmotropic ions and negative for chaotropic ions, and therefore, the Hofmeister series is usually constructed with the ions sorted according to their *B*-values (Figure 1).^13^

The Jones–Dole equation describes the viscosities of salt solutions up to 1 M well. We showed previously that the deviation from this expression of the more concentrated solutions can be explained by the presence of a critical concentration at which there are so many water molecules retained in the ion solvation layers that the system jams. This phenomenon is called the mayonnaise effect.^14^

The consequence of deviating from ideal, low-concentration solutions in the dynamics of water molecules is much debated. Classical molecular dynamics simulations show that ions influence the rotational dynamics of water molecules beyond their solvation layer.^16^ However, pump–probe spectroscopy measurements concluded that the addition of ions did not influence the rotational dynamics of water molecules outside the first solvation shell.^17,18^ These results were used to argue that the presence of ions does not lead to an enhancement or a breakdown of the hydrogen-bond network of bulk water, despite multiple spectroscopic experiments with X-ray,^19^ Raman,^20,21^ and neutron scattering^22^ showing perturbations in the bands associated with the hydrogen-bond network. Moreover, NMR is not very helpful when applied to ionic solutions because the average reorientation time of all of the water molecules is measured.^23^

Another source of disagreement is the extent of ion pairing and the homogeneity of concentrated salt solutions. The growing interest in water-in-salt solutions as potentially efficient and safe electrolytes for batteries has focused the discussion on the structure of the solutions when the concentration is so high that there are not enough water molecules to solvate the ions present. It has been proposed that these solutions have a homogeneous structure similar to that of molten salts, with a quasi-lattice governed by Coulombic laws, in which water molecules occupy interstitial or anion positions. This lattice would prevent hydrogen bonding between water molecules, as the average cation–anion distance does not allow a water molecule to associate with an anion without also associating with a cation.^24^ Molecular dynamics (MD),^25^ infrared (IR), ultrafast IR,^26,27^ and optical Kerr-effect (OKE)^28^ spectroscopy studies applied to the LiCl system at concentrations >10 M revealed that the vast majority of lithium ions form aggregates with the chlorine ions, such that the solution can be considered as a continuous network of water–ion structures. The interatomic distances of this network are similar to those determined by X-ray and neutron diffraction. These studies clearly indicate that hydrogen bonds between water molecules in these concentrated solutions are stronger than those in pure water and break and reform much more slowly. The existence of water molecule domains in these solutions is still under debate.

More than 50 years ago, a model of high, medium, and low ion concentration regions was proposed to explain the conductivity of saturated aqueous Ca(NO_3_)_2_ solutions, where, in the low-density structures, the ions are dispersed in a bulk water continuum.^29^ Such structures have been shown to exist in binary mixtures of aliphatic alcohols and water,^30−32^ where the proportion of water determines their size (on the order of nanometers), duration (between a few and hundreds of picoseconds), and ability to percolate, i.e., to form networks in which water molecules diffuse.^33^ These nanometric water structures have also been observed in eutectic solutions (6.76 M) of LiCl^34,35^ and LiCl with LiSCN.^36^ It remains to be investigated whether water aggregates also occur in solutions of other salts and concentrations. An initial NMR study has shown that a fraction of the water diffuses independently of the ions in saturated solutions.^37^ It has been found that in extremely concentrated solutions of lithium bis(trifluoromethane sulfonyl)imide (LiTFSI), the most common water-in-salt solute, the preference of water for lithium ions causes a solvation disproportionation that creates a nanoheterogeneous structure that immobilizes the anion and releases the Li^+^ from its Coulombic trap, allowing it to move much faster than would be expected at these concentrations^38,39^ in bulk-like water channels in which the water molecules act as a lubricant and a means of transport.^40−42^

**Figure 2:**
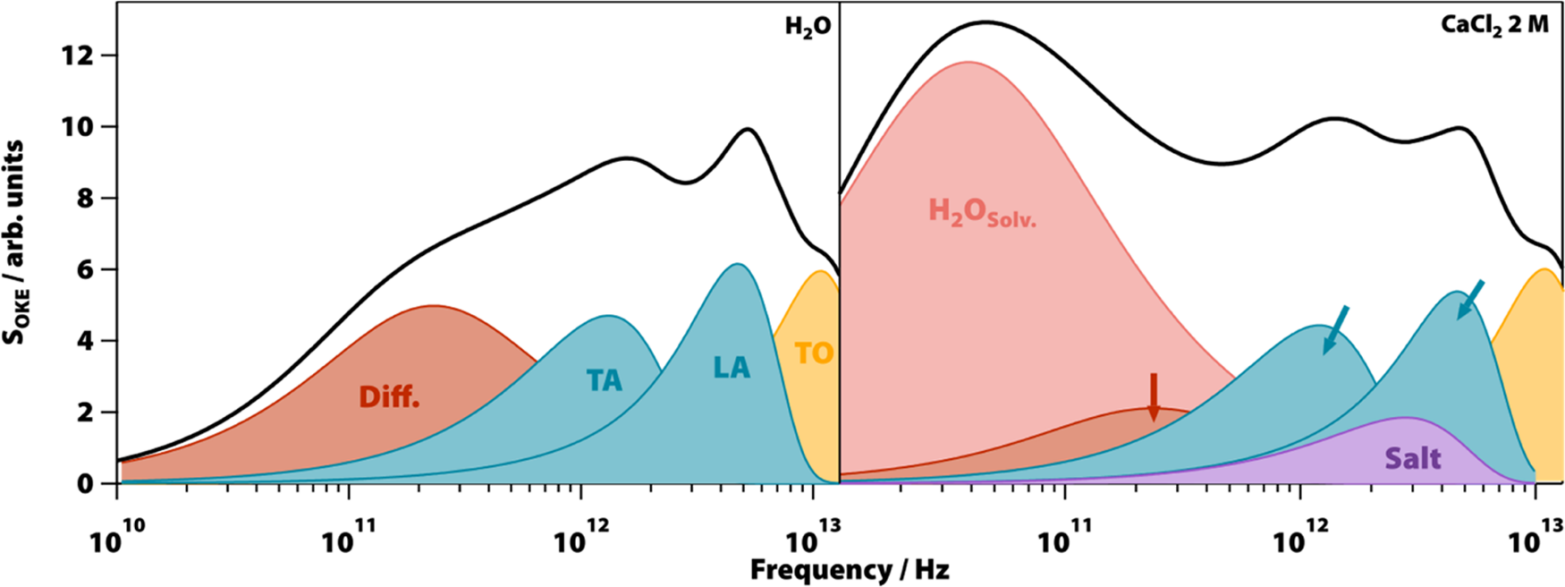
Analysis of the optical Kerr-effect (OKE) reduced-Raman spectra of water and aqueous solutions. (Left) Bands that compose the OKE spectrum of water. (Right) Changes that occur when a salt is added to water (CaCl_2_ 2 M). A band corresponding to the water in the solvation shell of the ion appears, while the band in pure water associated with the translational diffusion of its molecules weakens. The bands associated with the phonon-like modes change in intensity, and a new band appears in the terahertz region. This band is assigned to the saline aggregates that formed within the solution.

All of these contradictory results prevent a complete picture of the structure of water around ions, especially when they are chaotropic or at high concentrations. This is known as Hofmeister’s curse^6^ and must be overcome to understand the initial stages of nucleation and crystallization mechanisms as well as the role of water molecules in ion transport mechanisms in solutions such as water-in-salt electrolytes.^41^ Raman scattering spectroscopy can shed light on these discrepancies because it allows the study of different low-frequency dynamics separately. In the terahertz and subterahertz regions, water shows four distinct bands (Figure 2) associated with three different dynamical processes that can be studied by adding salts to the medium. From lower to higher frequencies, the first band, centered at 200 GHz, is associated with the diffusive translational dynamics of water molecules. The next two bands correspond to the restricted translation of water molecules within the liquid. One of these bands, with its maximum around ∼2 THz, is attributed to the O–O–O bonding of hydrogen bonds, and the other band, with its maximum around ∼5 THz, is related to O–O stretching along the O–H···O direction.^20^ These two bands can be described as transverse and longitudinal acoustic (TA and LA) phonon modes, respectively.^43^ The final band, centered at ∼13 THz, was associated with the librational twisting motion of water molecules in the liquid phase. Nevertheless, recent investigations have shown that, in order to reconcile the observed discrepancies between the Raman and infrared spectra, it must be assigned to a transverse optical (TO) phonon-like mode.^44^

Low-frequency spectra are difficult to study with Raman spectroscopy due to interference from a large Rayleigh scattering peak.^45^ Here, we avoid the problem by using femtosecond OKE spectroscopy, which measures the depolarized Raman spectrum in the time domain without the presence of a Rayleigh peak. This technique allowed us to study the effect of different cations and their concentrations on the low-frequency bands of water. OKE spectroscopy and terahertz-induced Kerr-effect spectroscopy have previously been used for this purpose.^26,28,46−49^ However, these experiments did not take into account the vibrational modes of the hydrogen bonds in most cases because they lacked sufficient temporal resolution to be sensitive to them. Our study is the first to separately analyze how hydrogen bonding and diffusion of water are altered by cations, providing insights into the ionic properties that alter the structure of water, both in the ion solvation layer and beyond.

## Results and Discussion

The OKE spectra of different chlorine salts in an aqueous solution were measured at 25 °C to study how cations alter the structure of water. The concentrations ranged from 0.5 M to the solubility limit. As indicated in the introduction, the charge and size of a cation are fundamental factors in its interaction with water. Therefore, a wide range of sizes and charges were investigated, including the chloride salts of all (nonradioactive) alkali metals, three alkaline earth metals (MgCl_2_, CaCl_2_, and BaCl_2_), lanthanum chloride (LaCl_3_), and ammonium chloride (NH_4_Cl). Hydrochloric acid (HCl aq) was also studied, although it is not a salt, because it dissociates completely in water, and so the effect of protons could be investigated. Chloride was used as the counterion because it has the smallest influence on the structure and dynamics of water. The *B*-coefficient of the Jones–Dole equation for chloride is almost zero (*B*^Cl^ = −0.005),^15^ and the residence time of water in the chloride hydration shell is 2.7 ps, which is very similar to that of water (3.4 ps).^50^

**Figure 3:**
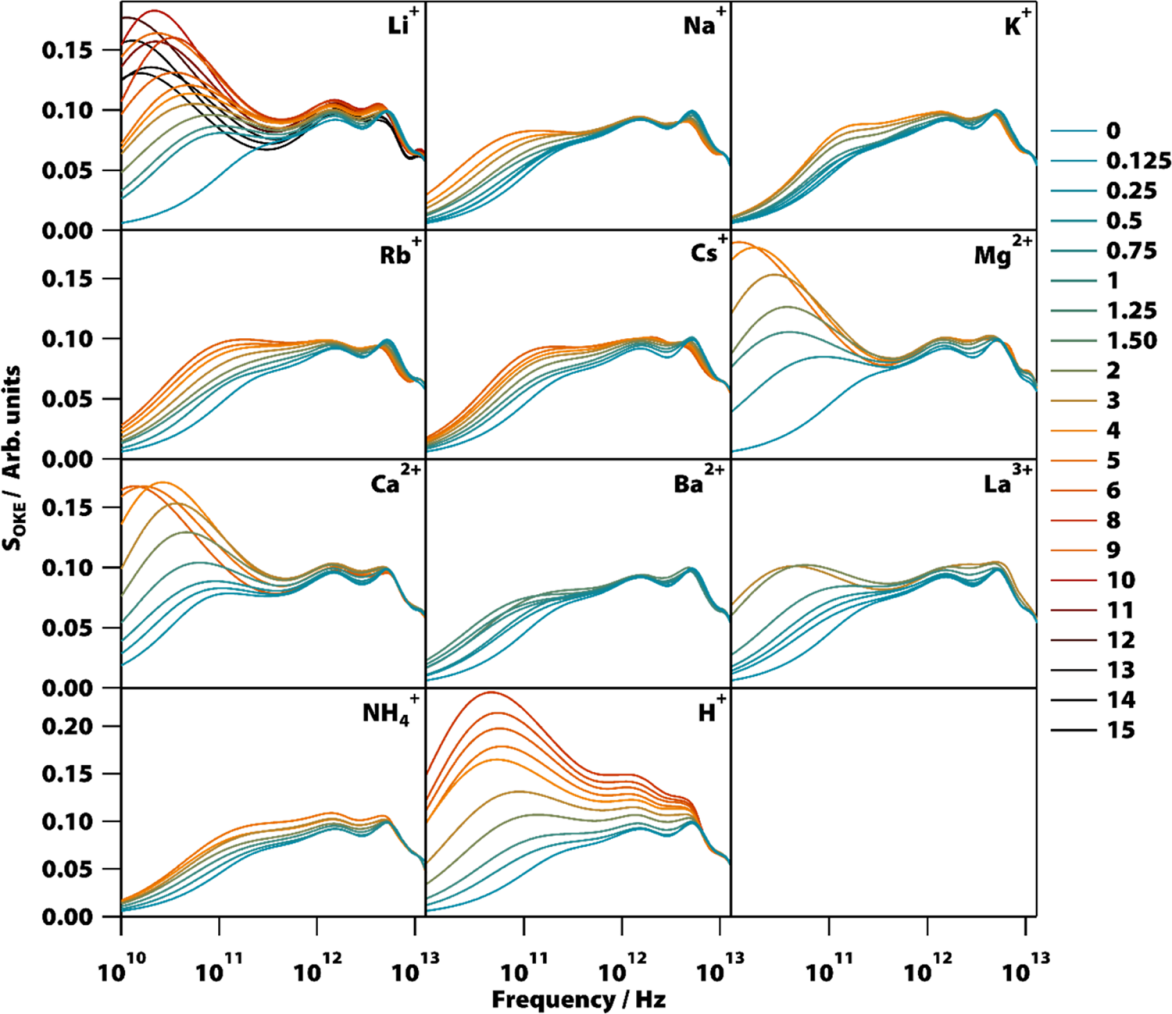
Experimental reduced-Raman OKE spectra of all of the measured salt solutions. Shown are the spectra of aqueous solutions of various metal chlorides at concentrations ranging from 0.125 to 15 M.

As each salt was added to water and its concentration increased, the spectra of the studied solutions generally exhibited the same changes (Figure 3): the portion above 10 THz remained relatively unchanged, while the lower-frequency part of the spectra became more intense and shifted to lower energy positions, and in the terahertz region, the two bands faded away as the intensity of the region increased.

The changes exhibited by the spectra of each solution were analyzed by using a model of water consisting of the following functions (Figure 2, left):

- One Cole–Cole function associated with the collision-induced diffusive translational motions of water. This function is a generalization of the Debye function that describes the relaxation of a material with a single relaxation time τ,
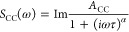
where *A*_CC_ is the amplitude of the function and α is the Cole–Cole exponent, whose value is between 0 and 1 and represents the inhomogeneity of the function.
- Two antisymmetrized Gaussian functions^51^ representing the phonon-like modes TA and LA,
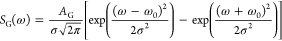
where σ is a width parameter.
- One Brownian oscillator to model the phonon-like TO mode
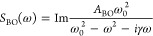
where ω_0_ is the undamped oscillator angular frequency and γ is the damping rate of the oscillation.

The parameters of each function for pure water at 25 °C were previously determined by fitting a set of water OKE spectra at a range of temperatures between 10 and 95 °C.^52^

As the concentration of the salts increased (Figure 2, right), the diffusional band of water strengthened and shifted toward lower frequencies (a second Cole–Cole function was needed to model this change), while the bands associated with the TA and LA phonon-like modes change their intensity and become less sharp. A third Gaussian function was used to account for the increase in the intensity in the space between these two bands. The band associated with the TO phonon-like mode hardly changed its appearance in the experiments, and in order to simplify the analysis, the parameters of the Brownian oscillator employed were kept constant. The following sections provide a detailed explanation of the changes that the model underwent during the experiments.

### Influence of Cations on Water Diffusion

The lower-frequency part of the water spectrum is typically attributed to the contribution of translational diffusion of water molecules.^43^ In principle, these dynamics should not be visible in Raman spectroscopy, but the collision of the molecules creates an induced anisotropy that makes them Raman-active.^34^ When salts are added to water, this band shifts to lower-frequency positions, and its intensity increases. This change is noticeable even at concentrations below 0.5 M, as shown by the spectra of sodium, potassium, and barium chloride in Figure 3. To date, the changes that salts cause in water diffusion have usually been interpreted as a collective increase in the inhomogeneity of the solvent.^35,53−55^

Here, however, a new approach to the problem is proposed. This approach assumes that there are two species of water in the solution: water molecules bound to neighboring water molecules by hydrogen bonds and water in the solvation shell around ions and ion aggregates. A Cole–Cole function was used to fit the contribution of the first species to the spectrum. The function kept the relaxation time and the Cole–Cole exponent (α) constant, and only its amplitude, *A*_CC_, was allowed to change. The parameters of the function in the spectrum of pure water at 25 °C were used as an initial guess.^52^ In all cases, the amplitude decreased as the salt concentration increased. The second species, water in the solvation shell around the ions, was also fitted with a Cole–Cole function. This band was more intense than the original diffusion band of pure water in all of the species studied. This is because the water molecules in the solvation shell are more polarized with the charges of the ions. The water molecules in the solvation shell also diffuse slower than bulk water molecules because of the stronger interaction with the ions (Figure 2).^56^

**Figure 4:**
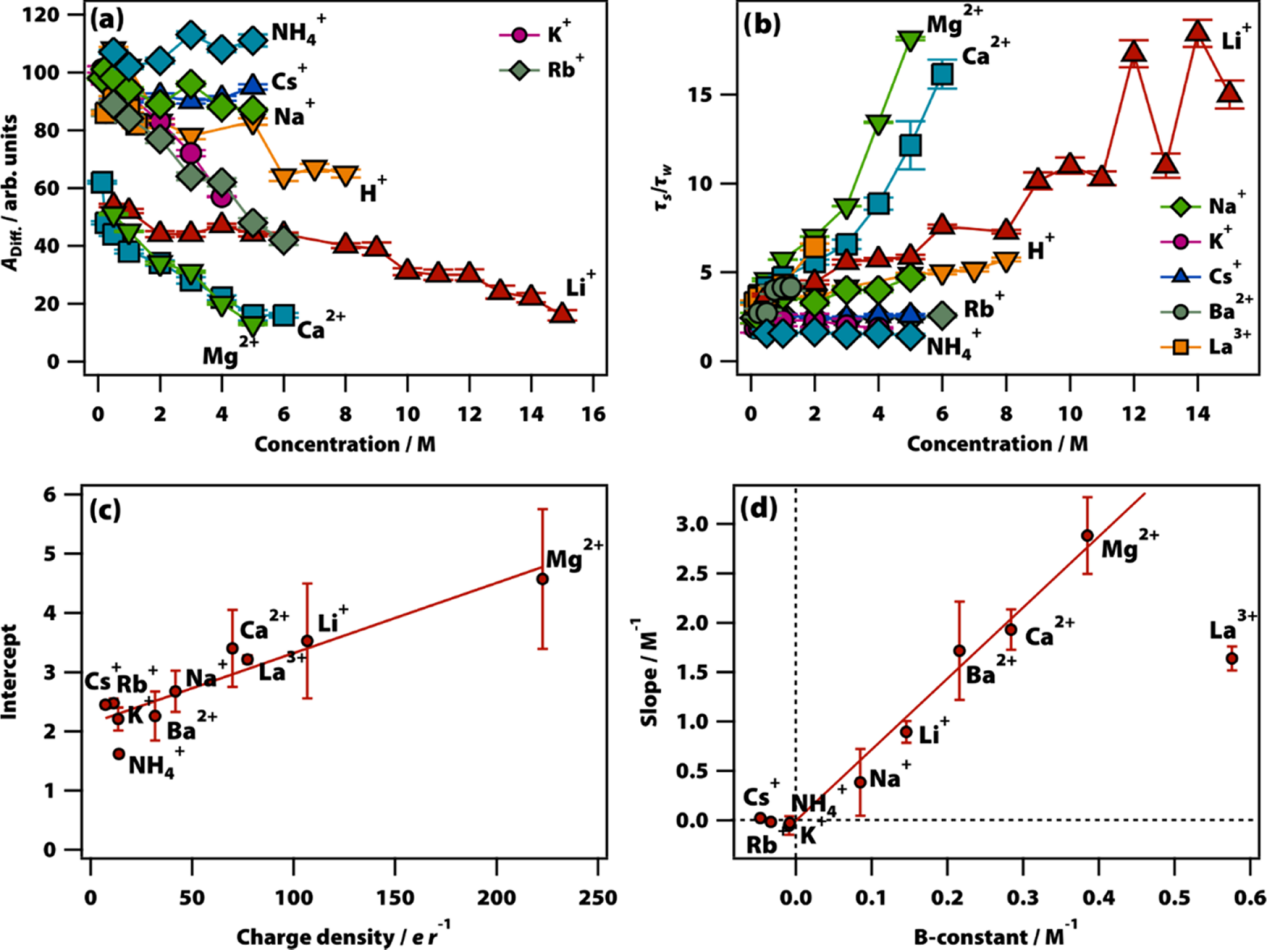
Effect of salt concentration on the diffusion of bulk-like water and water in the first solvation shell of cations. (a) Influence of the concentration on the amplitude (*A*_CC_) of the Cole–Cole functions associated with the water molecules bound to neighboring water molecules by hydrogen bonds. (b) Quotient of the relaxation times of the diffusion of water in the solvation shell of each cation (τ_s_) and the relaxation time of the translational diffusion of pure water (τ_w_) is plotted against the concentration of salt in the solution. (c) Correlation between the surface charge density of each cation and the intercept at zero concentration of their linear regression in panel (b). (d) Correlation between the *B* parameters of the kosmotropic cations^15^ studied and the slope of their linear regressions in panel (b). Linear regressions were performed on data with concentrations between 0 and 10 M.

The proposed model reveals that the band associated in pure water with the translational diffusion of its molecules (labeled as Diff. in Figure 2) decreases in intensity linearly as the salt concentration increases (Figure 4a). However, this band does not disappear even in solutions of cations with a high charge density (e.g., calcium, Figure S1) or high concentration (e.g., lithium, Figure S2). The persistence of this band in the most concentrated solutions, where the number of water molecules is insufficient to complete the coordination numbers of the ions, suggests that some water molecules still diffuse with a relaxation constant similar to that of pure water. This finding aligns with observations made in other highly concentrated water-in-salt solutions but deviates from the established understanding of concentrated lithium chloride solutions.^42^ Two-dimensional infrared (2D IR) experiments have demonstrated that the orientational relaxation of water molecules in >10 M LiCl solutions differs markedly from that in bulk water and the hydrogen bonds between water molecules are, on average, stronger and last longer than those in pure water.^26,27^ These experiments and molecular dynamics (MD) simulations^25^ suggest a continuous water–ion network structure where the formation of water clusters is highly improbable. However, the conclusions drawn from the experiments carried out in this work suggest otherwise. The 15 M LiCl solution, which is the most concentrated solution measured, still shows a weak band despite having only 2.5 water molecules per ion pair (mole fraction of χ_LiCl_ = 0.285). The change in amplitude with concentration shown in Figure 4a displays a trend that reinforces the interpretation of the band. Many MD studies demonstrate a high degree of ion clustering in solution, forming ion pairs and even polymeric species.^25^ This aggregation, together with the different solvation of Li^+^ and Cl^–^ ions (solvation disproportionation),^38^ would free enough water molecules to allow the formation of transient clusters of a few water molecules. These clusters do not have to be large to show bulk-like spectra, as MD simulations indicated that clusters as small as 5 water molecules show low-frequency Raman and IR spectra similar to that of bulk water.^57^ The dominance of TA and LA phonon peaks in the spectra at high concentrations, even at the saturation limit, reinforces the proposed interpretation as it was proved that they are signs of the presence of bulk-like water in the solution.^35,58^ This agrees with terahertz time-domain spectroscopy (THz-TDS) studies, which observed no dependence of the rotational relaxation time of water on the solute concentration in LiCl solutions.^59^ The proposed nanometer-sized water structures would be similar to the bulk-like water structures observed in binary mixtures of aliphatic alcohols and water,^30−32^ water-in-salt solutions of concentrated LiTFSI,^38,42,60^ and eutectic lithium chloride solutions.^35,58^

Clustered water molecules are just one element of the vast array of water molecules found in a saturated salt solution, where they take all kinds of positions and roles around and between the free ions, ion pairs, and larger aggregates of ions present in the mixture. The proposed model reflects this diversity of environments in the band associated with the water molecules in the ion solvation shell. The observed correlation between decreasing Cole–Cole exponent (α) and increasing concentration in all of the studied salts (Figure S3) supports this interpretation, providing evidence for the increased complexity and heterogeneity of the relaxation processes of the water molecules in solution. The relaxation times of water molecules in the ion solvation shell (τ_s_) and in pure water (τ_w_) can be used to determine the average slowing of water molecules as they enter into the ion solvation shell. By calculating the ratio τ_s_/τ_w_ for each ion at all measured concentrations, a linear relationship between the slowing down and the ion concentration can be observed (Figure 4b). A linear regression of each of these lines provides important information about the influence of the ions on the water molecules. The point where the regression line intersects the axis where *c* = 0 gives the slowing down value of an ideal solution. This value represents the slowing that water molecules would undergo on an ion surface without the interference of other surrounding ions. Figure 4c shows that this slowing down depends on the charge density of the cation.

However, as the cation concentration increases and bulk-like water and solvated ion domains start to form, charge density is no longer the influencing factor. Figure 4d shows that the slope of the ratio τ_s_/τ_w_ vs concentration lines, i.e., the influence of increasing concentration on the slowing down of the solvated water dynamics, depends on the chaotropic or kosmotropic capacity of the cations. The only cation that does not follow this trend is La^3+^, perhaps because at high concentrations, the LnCl_3_ salt is not completely dissociated.^61^

For the kosmotropic cations (*B* > 0), as their concentration in the solution increases, the slowdown increases linearly with the *B*-coefficient of the Jones–Dole equation. In contrast, for the chaotropic (*B* < 0) cations studied, the increase in their concentration does not change the slowdown suffered by the water molecules. This lack of influence of the *B*-parameter, or, in other words, of the interaction of the chaotropic ions with their environment, explains the abnormality observed in these ions when they require a higher critical concentration than expected according to the mayonnaise effect. This result also brings a new aspect to the Hofmeister scale. These results also reveal that the correlation between water relaxation time and viscosity that has been previously observed is actually due to the properties of water in the cation solvation layer.^62^

### Influence of Cation on the Hydrogen-Bond Network

The addition of salts to water causes changes in the two Gaussian functions used to model the TA and LA vibrations of the water. The bands widen, shift to lower frequencies, and (except for hydrochloric acid) become less intense as the salt concentration increases (Figure 2). These changes are similar to those that occur when pure water is heated^52^ and suggest that the hydrogen bonding network of the medium is weakening.^20^

**Figure 5:**
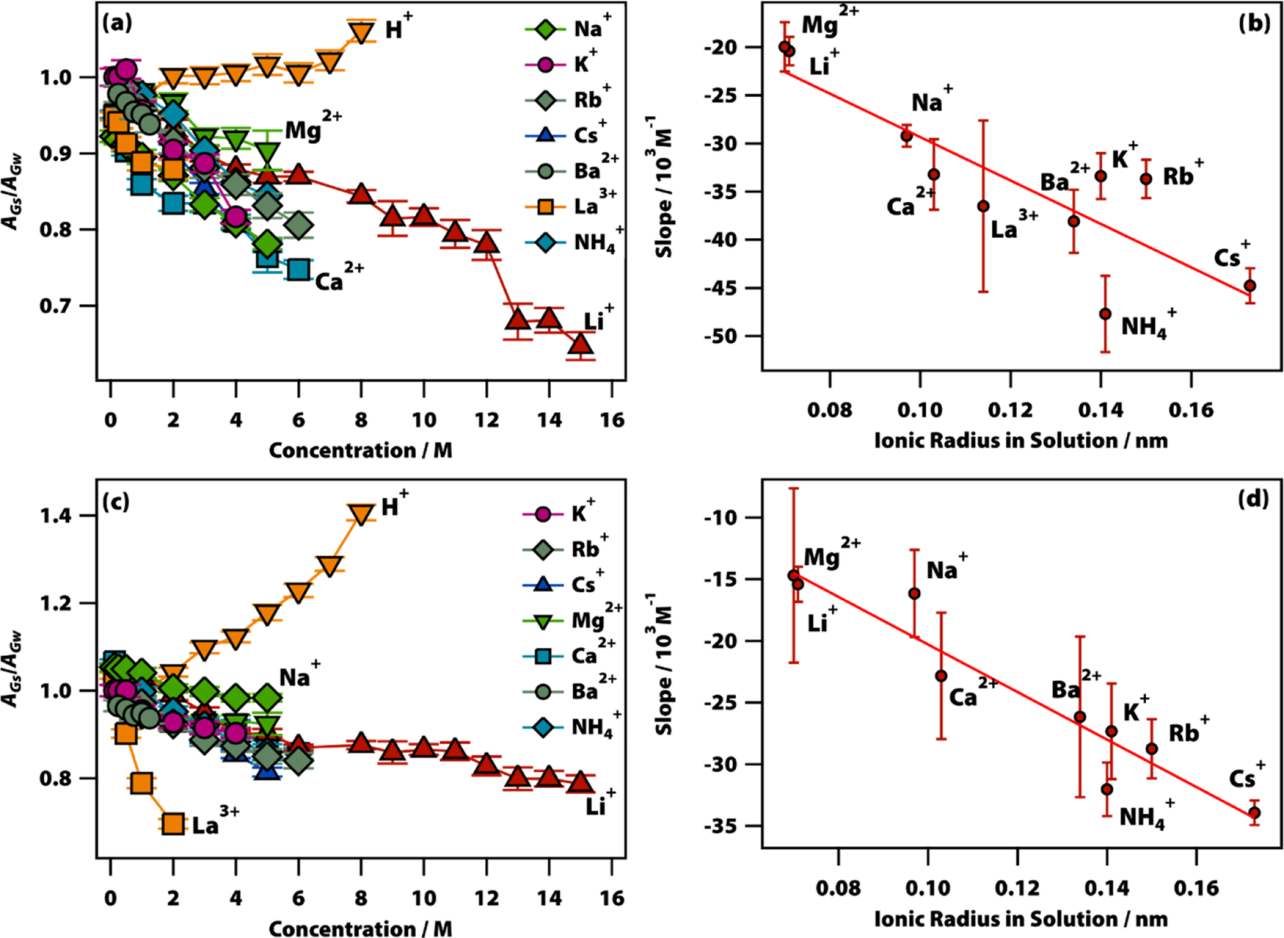
Effect of salt concentration on the hydrogen-bond associated acoustic-phonon bands of water. The results for the TA band are shown on top and that of the LA band at the bottom. (a) Quotient of the intensity of the Gaussian associated with TA band (*A*_Gs_) and the same band in pure water (*A*_Gw_) is plotted against the concentration of salt in the solution. (b) Correlation between the ionic radius in solution of the cations and the slope of their linear regressions in Figure 4a. (c) Quotient of the intensity of the Gaussian associated with LA (*A*_Gs_) and the same band in pure water (*A*_Gw_) is plotted against the concentration of salt in the solution. (d) Correlation between the ionic radius in solution^64^ of the cations and the slope of their linear regressions in Figure 4c. Linear regressions were performed on data with concentrations between 0 and 12 M.

To determine how each cation affects the TA and LA bands, we plotted the ratio of the amplitude of the antisymmetrized Gaussian functions of the solutions (*A*_Gs_) to the amplitude of the same function in pure water (*A*_Gw_) vs the cation concentration. This revealed a linear trend between cation concentration and the change in the intensity of the bands (Figure 5a for the TA band and Figure 5c for the LA band). The slopes of these plots can be used to quantify the effect that each cation has on the hydrogen-bond structure of water.

Hydrochloric acid was the only species that showed positive slopes in the TA and LA bands. This means that increasing the concentration of protons in solution enhances the hydrogen-bond network of water by increasing the number of O–O bonds.^63^ The rest of the cations show negative slopes. The slopes are steeper in the TA bands. Some of them, like La^3+^, Na^+^ and Ca^2+^, show at concentrations below 2 M a strengthening of the TA band, but this distinct reinforcement of the water structure does not depend on the B-parameter of the cations and therefore is not related to their kosmotropic properties.

The slopes in Figure 5a,c do not depend on the charge density or the B-coefficient of the cations. However, a correlation between the ionic radius in solution and the slopes was observed for both the TA and LA bands. The ammonium ion deviated from the general trend, suggesting that the size factor is more complex. The lanthanum ion also deviated in the LA band from the general trend. Therefore, cations do have an important effect on hydrogen bonds, which is independent of their ability to break or form structures and depends on their size.

### Appearance of a Band Associated with Saline Aggregates

The disappearance of the valley between the TA and LA bands in water, as the concentration of salt in the solution increases, cannot be explained by the changes in the intensity, position, or shape of these bands. It is necessary to use at least one more function to model this feature (Figure 2, right). The fact that the intensity of this feature increases with an increasing solute concentration suggests that it is related to aggregates of hydrated ions. The position of this new band supports this assignment, as it appears in the frequency range of phonons in the crystals of the salts between cations and chlorine (Figure S4). However, it is not possible to fit in a simple way the growth of this feature with a number of functions that represent different types of ionic aggregates (e.g., outer- and inner-sphere ion pairs, for example). The fitting of this feature was performed using a broad antisymmetrized Gaussian function.

The fit of the Gaussian function shows that the intensity of the band in the different salts generally increases with concentration, following a saturation curve (Figure S5). This is consistent with the equilibria necessary to form a saline aggregate and supports the interpretation of the band origin as being due to such aggregates. These aggregates, which, according to measured spectra, can form at relatively low concentrations, may be a first step in nucleation and crystallization processes.^65,66^

## Conclusions

Optical Kerr-effect (OKE) spectroscopy can be used to examine the low-frequency dynamics of water molecules in the presence of various cations. By fitting the OKE spectra, it is possible to determine how the concentration and properties of the cations alter the translational diffusion of water molecules and the hydrogen-bond structure of the solution. The diffusional part of the spectrum reveals two bands: a slower band, associated with water in the ion solvation shell, and another band with the same properties as pure water, associated with the molecules of water binding to neighboring water molecules by hydrogen bonds. This demonstrates that the ions only slow down the translational dynamics of the water molecules in the ion solvation layer but do not alter the dynamics of bulk-like water molecules, contrary to what numerous studies have argued.

The slowdown of water molecules in the solvation shell under infinite dilution conditions depends on only the charge density of the cation. However, as the salt concentration increases and the interactions between the ions become significant, the cations slow down the water molecules, following the Hofmeister series. Chaotropic cations have a minimal effect, while kosmotropic cations increase the slowdown, depending on their B-coefficient from the Jones–Dole equation.

The existence of a band associated with water molecules that bond to neighboring water molecules by hydrogen bonds even at the highest salt concentrations, when, in salts such as LiCl, there is insufficient water to solvate all of the ions, implies that the solution has a heterogeneous structure composed of nanometer-sized water clusters and salt aggregates. This is additionally supported by the appearance of a new band in the spectrum whose intensity depends on the salt concentration and appears in the frequency range of crystal phonons. This result is important for the study of the initial stages of solute nucleation and crystallization mechanisms as well as facilitating a deeper comprehension of the models that explain the transport of cations in the water-in-salt electrolytes used in batteries. The observed band can be used for further study of said processes.

Because the presence of ions does not affect bulk-like water diffusion in diluted solutions, it has been suggested that ions do not alter the overall hydrogen bonding. Nevertheless, it was found that the addition of salts reduces the strength of the TA and LA phonon bands linked to hydrogen-bond vibrations. This weakening intensifies as the concentration increases and depends on the size of the cation. Hydrochloric acid is an exception, as it strengthens the hydrogen-bond network by increasing the number of the O–O bonds.

## Supporting Information Available

The experimental methods of this work, all measured OKE spectra of lithium chloride and calcium chloride with their respective fits, the Raman spectra of crystalline LiCl, and a figure showing the influence of concentration on the intensity of the band associated with the salt aggregates (PDF)
