# Supplementary material for: Lifting Hofmeister’s Curse: Impact of Cations on Diffusion, Hydrogen Bonding, and Clustering of Water
Source: J Am Chem Soc. 2023 Dec 21;146(1):368–76. doi: 10.1021/jacs.3c09421 (PMC10786029; doi:10.1021/jacs.3c09421)
Supplement: Supplementary file 1 — ja3c09421_si_001.pdf [file ja3c09421_si_001.pdf]

Supporting Information for Publication
Lifting Hofmeister's curse: Impact of cations on diffusion, hydrogen bonding and clustering of water

Mario González-Jiménez, Zhiyu Liao, Elen Lloyd Williams, Klaas Wynne
School of Chemistry, University of Glasgow, UK

EXPERIMENTAL METHODS

The OKE spectra were recorded in a standard time-domain step-scan pump-probe configuration and Fourier transformed to obtain the frequency-domain reduced depolarized Raman spectrum as described previously. A laser oscillator (Coherent Micra) provided 10-nJ pulses (0.8 W average power) with a nominal wavelength of 800 nm at a repetition rate of 82 MHz, resulting in a 20-fs pulse width in the sample. Pump-probe OKE experiments were carried out with delay times from a few femtoseconds to a maximum of 100 ps, resulting in a spectral resolution of better than 10 GHz (0.33 cm-1). The sample was contained in a rectangular quartz cuvette (Starna, thickness: 1 mm) held in an aluminum block that was temperature-controlled to a precision of ± 0.1 °C.

All the chemicals were purchased from Sigma-Aldrich except for $LaCl_3 \cdot 7\ H_2O$, which was purchased from Alfa-Aesar. All of them had a purity of over 99% and were used without any further purification. The water used was HPLC gradient grade (Fisher Chemical). Solutions were filtered using 0.2 mm hydrophilic polytetrafluoroethylene (PTFE) filters (Millipore) to remove dust before their transfer to the cuvette.

The error bars in Figures 4 (a), 4(b), 5(a), 5(c), and S4 represent the 95% confidence interval for the uncertainty in the parameter value. This interval was calculated by simultaneously fitting all the parameters of the function used to model the band they belong to, while keeping constant all the parameters of the rest of the functions used to fit the spectrum. In the remaining figures, the error bars denote the standard error resulting from the linear regression employed to obtain the value of each parameter.

SUPPLEMENTARY FIGURES

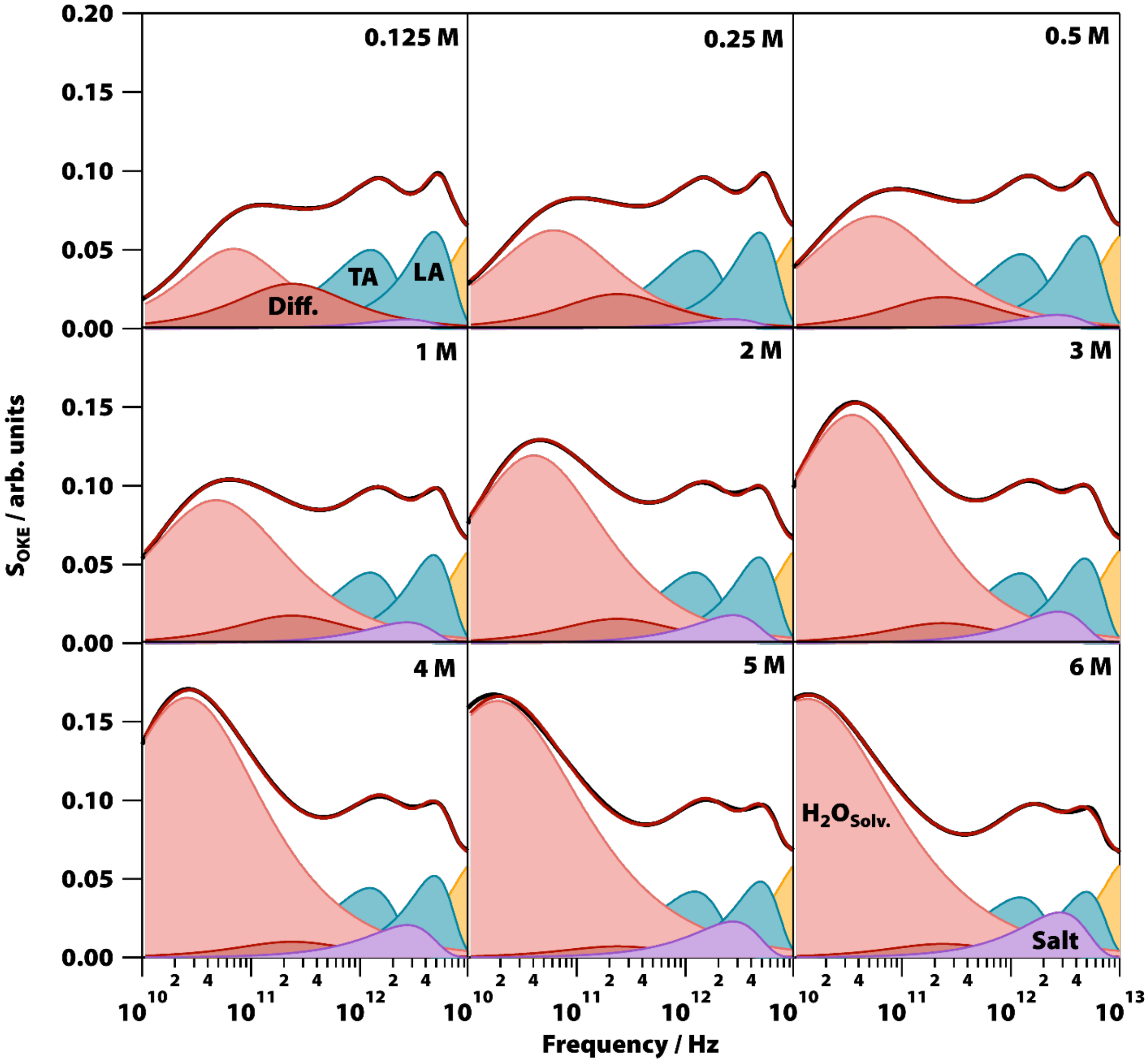

Figure S1. OKE spectra (black) and fit (red) of all the aqueous calcium chloride solutions measured. The functions used for each model are also shown. Note the presence of a bulk water band in the spectra with the highest salt concentrations.

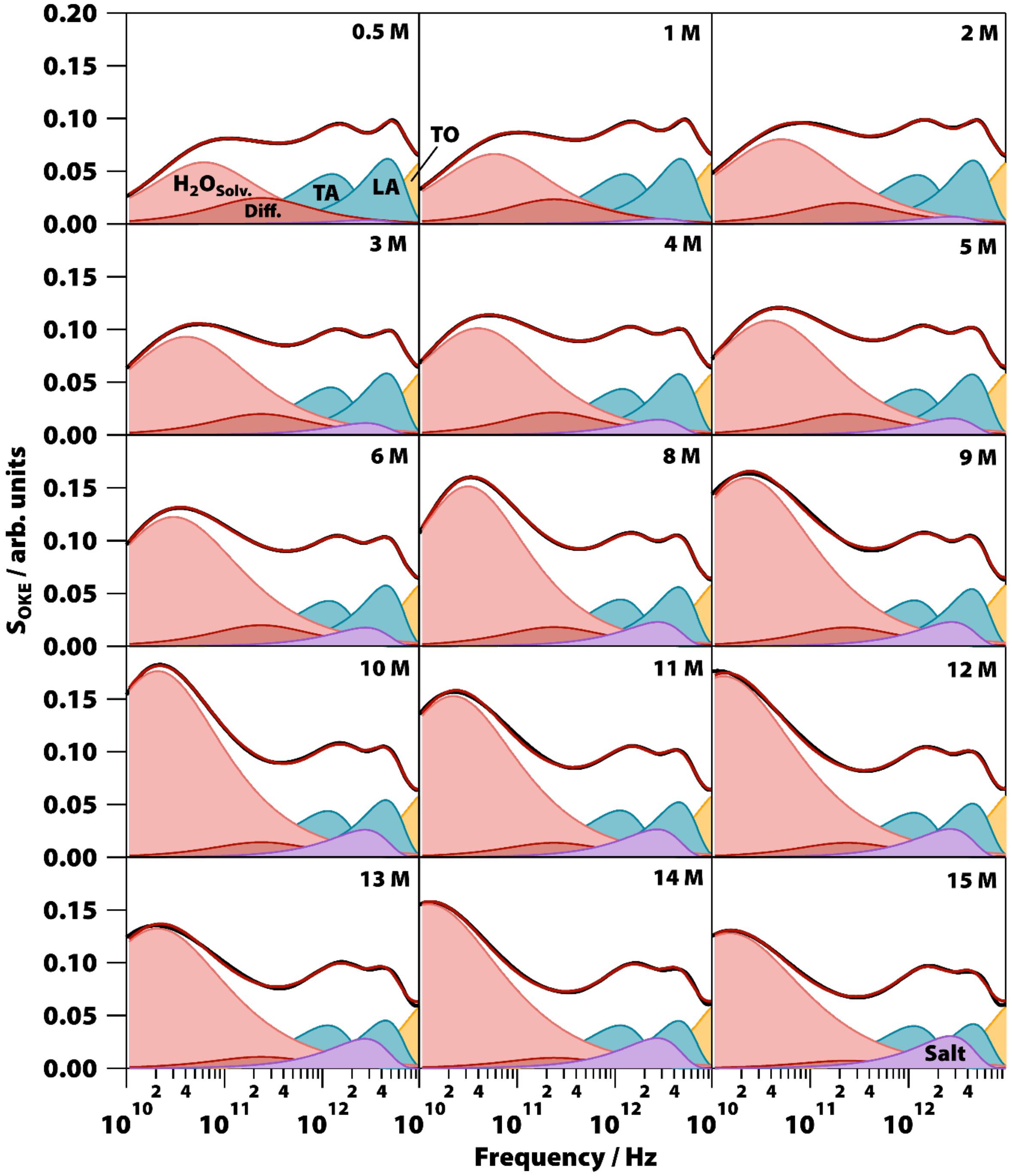

Figure S2. OKE spectra (black) and fit (red) of all the aqueous lithium chloride solutions measured. The functions used for each model are also shown. Note the presence of a bulk water band in the spectra with the highest salt concentrations.

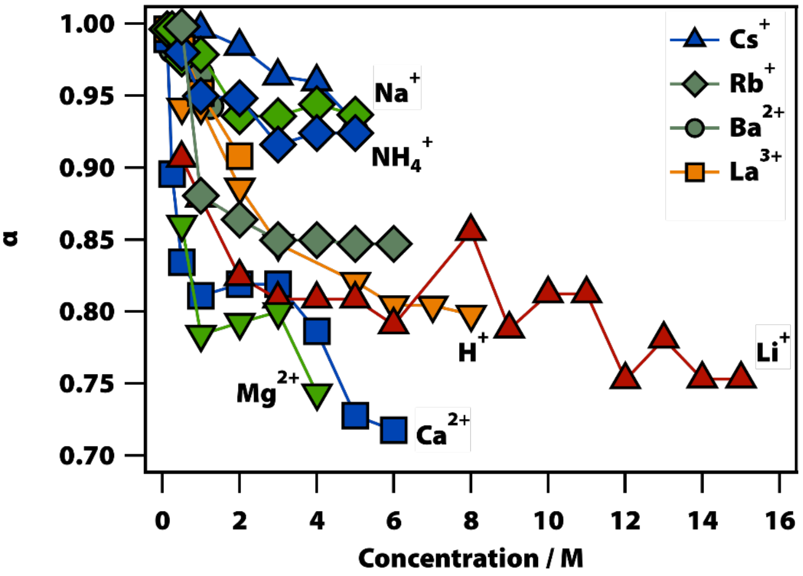

Figure S3. Influence of the salt concentration on the value of the Cole-Cole exponent (α) of the Cole-Cole function associated with the collision-induced diffusive translational motions of water in the solvation shell of each cation.

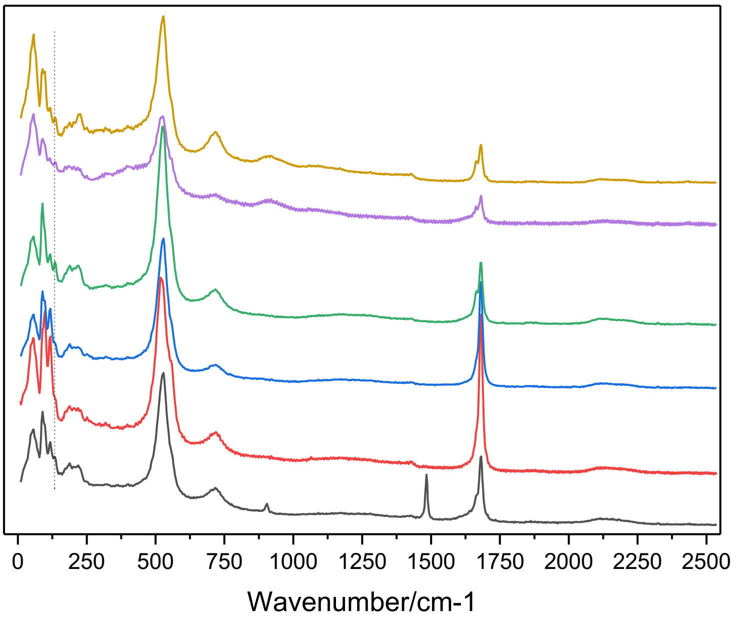

Figure S4. Raman spectra of LiCl crystalline. The phonon bands of the crystal appear around 60 cm^{-1} (1.7 THz), which is in the range of frequencies where the saline aggregates appear.

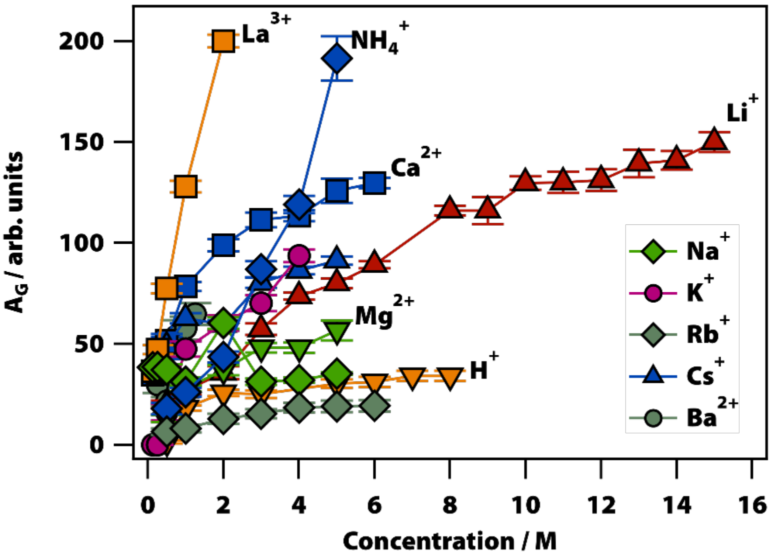

Figure S5. Concentration dependence of the amplitude (A_G) of the Gaussian functions associated with the aggregates of each salt. The saturation curve profile supports this assignment.
